# Examining covariate-specific treatment effects in individual participant data meta-analysis: Framing aggregation bias in terms of trial-level confounding and funnel plots

**DOI:** 10.1017/rsm.2025.10043

**Published:** 2025-10-23

**Authors:** Lianne K. Siegel, Joseph S. Koopmeiners, Jamie Hartmann-Boyce, Peter J. Godolphin, Abdel G. Babiker, Giota Touloumi, Kirk U. Knowlton, Richard D. Riley

**Affiliations:** 1 Division of Biostatistics and Health Data Science, School of Public Health, University of Minnesota, Minneapolis, MN, USA; 2 Department of Health Promotion and Policy, School of Public Health & Health Sciences, University of Massachusetts Amherst, Amherst, MA, USA; 3 MRC Clinical Trials Unit at UCL, University College London, London, UK; 4 Department of Hygiene, Epidemiology and Medical Statistics, Medical School, National and Kapodistrian University of Athens, Athens, Greece; 5 Intermountain Medical Center, Intermountain Heart Institute, Salt Lake City, UT, USA; 6 School of Health Sciences, University of Birmingham, Birmingham, UK; and National Institute for Health and Care Research (NIHR) Birmingham Biomedical Research Centre, UK.

**Keywords:** aggregation bias, heterogeneity, individual participant data, interactions, IPD, meta-analysis

## Abstract

To understand a treatment’s potential impact at the individual level, it is crucial to explore whether the effect differs across patient subgroups and covariate values. Meta-analysis provides an important tool for detecting treatment–covariate interactions, as it can improve power compared to a single study. However, aggregation bias can occur when estimating individual-level treatment–covariate interactions in meta-analysis, due to trial-level confounding. This refers to when the association between the covariate and treatment effect *across* trials (at the aggregate level) differs from that observed *within* trials (at the individual level). It is, thus, recommended that heterogeneity in the treatment effect at the individual level should be disentangled from that at the trial level, ideally using an individual participant data (IPD) meta-analysis. Here, we explain this issue and provide new intuition about how trial-level confounding is impacted by differences in within-trial distributions of covariates and how this corresponds to asymmetry in subgroup-specific funnel plots in the case of categorical covariates. We then propose a sensitivity analysis to assess the robustness of interaction estimates to potential trial-level confounding. We illustrate these concepts using simulated and real data from an IPD meta-analysis of trials conducted on the TICO/ACTIV-3 platform, which assessed passive immunotherapy treatments for inpatients with COVID-19.

## Highlights

### What is already known?

- Individual participant data meta-analysis is an important tool for examining individual-level interactions that single studies may lack sufficient power to detect.
- Aggregation bias can arise when estimating treatment–covariate interactions when within- and between-study information are not disentangled.
- Many IPD meta-analyses still do not separate within and between-study information in practice.

### What is new?

- We reframe aggregation bias as trial-level confounding and explain its source differently than previous work.
- Trial-level confounding can be observed as subgroup-specific funnel plot asymmetry.
- We propose a simple sensitivity analysis to assess the robustness of results to trial-level confounding.

### Potential impact for RSM readers

- Practitioners will be able to recognize better and address potential sources of confounding when estimating treatment–covariate interactions in an individual participant data meta-analysis.

## 1 Introduction

It is important to explore heterogeneity in the effect of a new treatment across patient subgroups defined by individual-level covariate values to fully understand its potential impact.^1^ This requires examining whether there is an interaction between the treatment effect and the covariate(s) of interest, such as when the treatment effect is different for males and females.^2^ Any observed interaction can then be further interpreted by estimating covariate-specific treatment effects. These may consist of several subgroup-specific treatment effects for a categorical covariate, or a treatment-effect curve for a continuous covariate.^3^^,^ ^4^ However, most randomized trials, the gold standard for assessing causal treatment effects, are underpowered to detect treatment–covariate interactions. Meta-analysis can, therefore, increase power for detecting treatment–covariate interactions that may not be evident from a single trial.^5^^,^ ^6^

**Figure 1:**
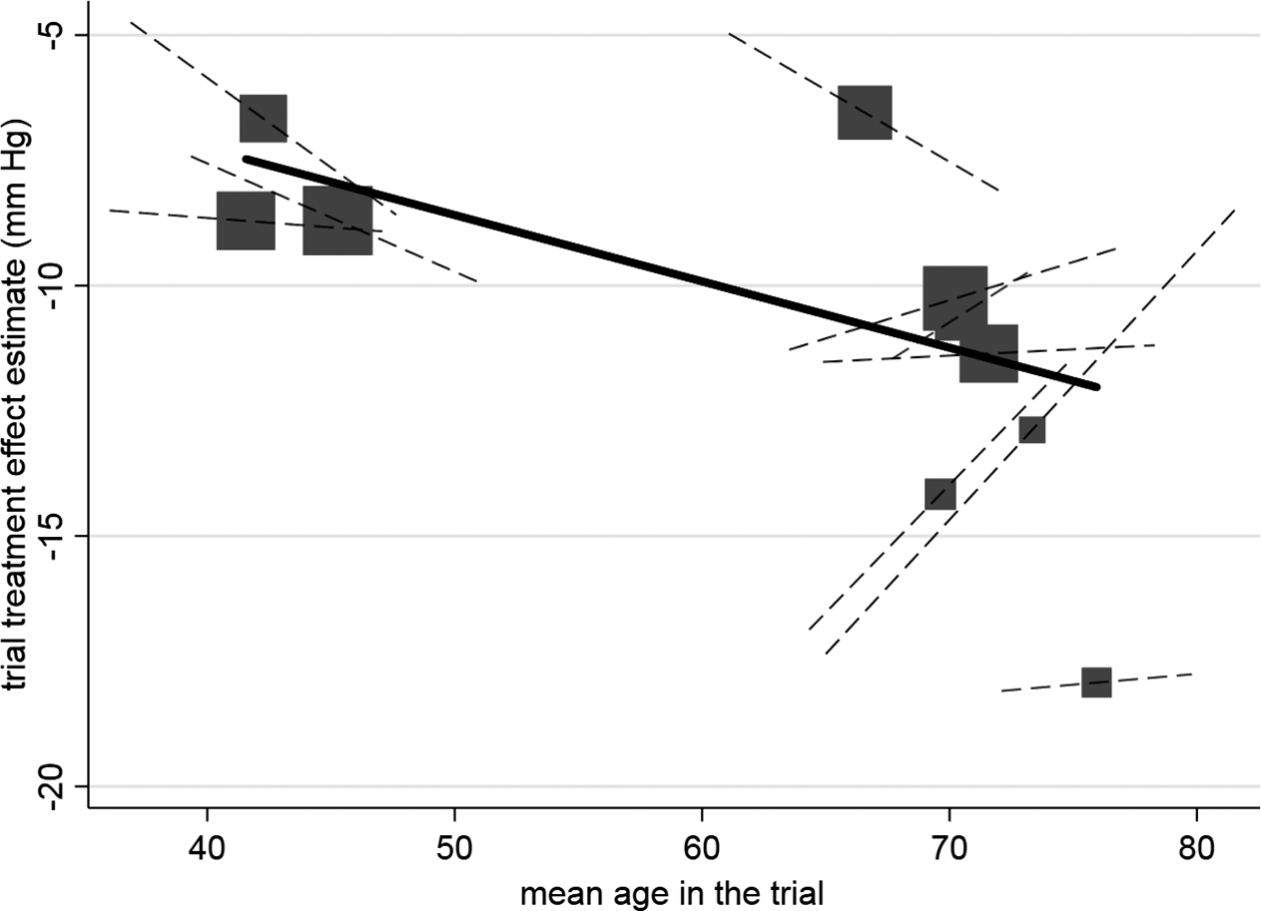
Example of aggregation bias when estimating the individual-level interaction between age and the effect of hypertension treatment. Across-trial relationship (from meta-regression of trial-level treatment effect estimates versus mean age), denoted by gradient of solid line, which is statistically significant. Participant-level relationship using within-study information (i.e., treatment–age interaction within each trial) denoted by gradient of dashed lines, and their average gradient is −0.036 (95% CI: −0.19 to 0.12). Each block represents one trial, and the block size is proportional to the size of the trial. Reproduced from Figure 2 in Riley et al.^3^ (CC-BY license).

Ideally, individual-level heterogeneity in the treatment effect should be estimated while avoiding aggregation bias, also referred to as the ecological fallacy.^7^^,^ ^8^ Aggregation bias arises when amalgamating incompatible within-study and across-study information; specifically, when the across-study association between study-level treatment effects and covariates aggregated at the study level (e.g., mean age) does not align with the within-study association at the individual level between individual-level treatment effects and covariate values^7^^,^ ^9^^,^ ^10^ (Figure 1). It is, therefore, recommended to disentangle the two sources of information by using an appropriate individual participant data (IPD) meta-analysis (MA) model,^11^ or by extracting and pooling treatment–covariate interactions at the individual level from existing studies.^3^^,^ ^4^^,^ ^7^^,^ ^11^^–^ ^13^ The same issue has been raised in other settings involving clustering, including multicenter trials.^14^

However, many practitioners do not separate within- and between-study information when estimating treatment–covariate interactions or explain why they opted not to.^7^ For example, a recent review of 100 IPDMAs found that only 15% stated doing so.^15^ We hypothesize that this may be because practitioners overlook that aggregation bias can occur despite randomization, and because estimating individual-level covariate-specific treatment effects requires more complex statistical models that separate within- and between-study information.

To address this, we frame aggregation bias as a form of trial-level confounding, rather than an inconsistency between within- and across-trial information, and explain how it can be assessed using existing meta-analytic tools. We first demonstrate how trial-level confounding occurs in IPDMA when using across-trial information, either when (1) the treatment effects differ across studies and are associated with the distribution of the covariate, or (2) the prognostic effect of the covariate differs across studies and is associated with the treatment allocation ratio. We then demonstrate how trial-level confounding is reflected in subgroup-specific funnel plot asymmetry and models that avoid it. To address the lack of power to test for the presence of aggregation bias,^10^^,^ ^16^ we propose an alternative sensitivity analysis approach.

## 2 Motivating example: IPDMA of passive immunotherapy for COVID-19

The TICO (Therapeutics for Inpatients With COVID-19)/ACTIV-3 master protocol^17^ evaluated five investigational agents providing passive immunotherapy targeting the SARS-CoV-2 virus among hospitalized patients.^18^^–^ ^21^ None of the agents were shown to increase the rate of the primary outcome of 90-day sustained recovery and all but one trial were stopped after a pre-specified futility analysis. Methods and results of these trials have been previously reported.^18^^–^ ^21^ While none of the five trials were powered to investigate treatment–covariate interactions, there was an a priori hypothesis that the treatment effect would be greater among participants negative for SARS-CoV-2 neutralizing antibodies (seronegative) at baseline. Thus, despite no evidence of an overall effect, there may still be benefit in the subgroup of seronegative participants. Knowlton et al.^22^ examined this potential interaction in an IPD meta-analysis, which also included the ITAC trial of hyperimmunoglobulin therapy.^23^ We note that the enrollment periods for several of the TICO/ACTIV-3 trials overlapped; participants who were concurrently eligible to receive multiple agents were first randomized to a specific active agent or its matched placebo. Participants randomized to placebo were then shared across trials for which they had been eligible.^17^ We consider a participant randomized within a specific trial if they were assigned that active agent or its matched placebo.

## 3 One-stage versus two-stage approach to IPDMA

An IPDMA is typically conducted using either a one-stage or two-stage approach to summarize the effect of interest, such as an overall treatment effect or a treatment–covariate interaction.^24^^,^ ^25^ A one-stage approach involves fitting a single model (e.g., generalized linear mixed model) directly using the IPD while accounting for clustering of individuals within trials; thus, estimating trial-specific and meta-analysis parameters simultaneously. Alternatively, a two-stage approach involves first fitting trial-specific models (e.g., regressions) using the IPD within each trial separately, then the trial-specific estimates (e.g., regression coefficients) are pooled using a separate meta-analysis model to estimate the overall effect of interest.^26^ If multiple coefficients from each trial are of interest, multivariate meta-analysis can be used to pool them simultaneously and estimate their joint distribution.^3^ Either approach can assume that the trials share the same underlying effect of interest, as in a common (fixed) effect meta-analysis, or that there is heterogeneity in the underlying coefficients, as in a random effects meta-analysis that assumes particular trial-specific coefficients (e.g., treatment–covariate interaction) are exchangeable (e.g., following a normal distribution).^27^ Extensive previous work has considered the choice of one-stage versus two-stage methods; this has shown that the results of both approaches tend to be very similar as long as the same estimation methods and modeling assumptions are used, unless the sample size is small or the outcome is rare.^11^^,^ ^26^^,^ ^28^

## 4 Sources of trial-level confounding: Simulated data motivated by TICO/ACTIV-3

### 4.1 Model assuming common main effects of treatment and covariate

Suppose that following a one-stage approach, a logistic regression model is fit directly on the pooled IPD. The model consists of separate fixed intercepts ( 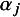 ) for each trial ( 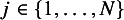 , where *N* is the total number of trials in the meta-analysis), a common main effect ( 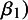 of treatment assignment 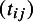 , a common main effect ( 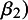 of SARS-CoV-2 neutralizing antibody seropositivity) 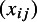 , and a single term for the effect of the interaction ( 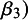 between baseline seropositivity and the treatment assignment for the participant 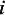 in trial 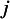 :(1)

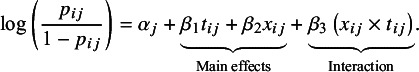



The separate trial-specific intercepts allow for different baseline (control group) mortality rates across trials, but the other covariate effects are implicitly assumed to be common. This model does not separate within-trial from across-trial information, because the interaction term can explain both within-trial and across-trial heterogeneity in the treatment effect when 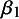 and 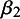 are not stratified by study.^7^ Thus, we will demonstrate how Model (1) is still at risk of aggregation bias due to trial-level confounding despite directly using IPD.

**Figure 2:**
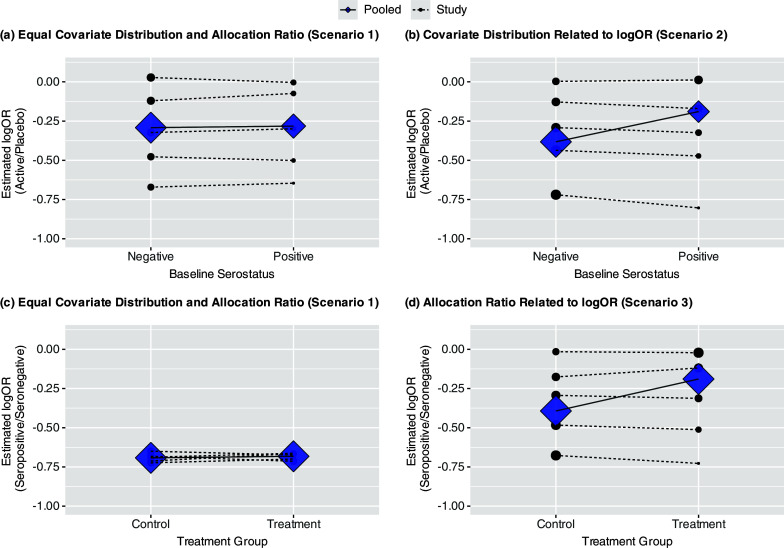
(a–d) Four panels showing potential for aggregation bias under three scenarios: no trial-level confounding (Scenario 1), trial-level confounding by covariate (Scenario 2), trial-level confounding by allocation ratio (Scenario 3). Trial-specific estimated log ORs given by solid dots; diamonds represent estimated subgroup-specific pooled effects from Model (1); the sizes of these are proportional to the inverse of their variances. Within-trial and pooled estimated interactions are given by dashed and solid lines, respectively. (a–b) show estimated log OR comparing treatment versus placebo by baseline serostatus; (c–d) show estimated log OR comparing seropositive versus seronegative (prognostic effect) by randomization group. In all cases, the slope of the lines represents the coefficient for the interaction between treatment group and baseline serostatus.

### 4.2 Data-generating mechanism

To illustrate potential sources of confounding, we simulate data from five trials under three hypothetical scenarios motivated by TICO/ACTIV-3. As a more general case, these trials are generated separately with no sharing of participants assigned to placebo across trials. We conduct IPDMAs of the simulated data assessing whether the treatment effect of passive immunotherapy in hospitalized patients with COVID-19 on a binary outcome of mortality differs between participants positive or negative for SARS-CoV-2 neutralizing antibodies at baseline. A common (fixed) effect approach is used, which estimates a common treatment–covariate interaction from the five trials. In each scenario, we generated a single dataset with 100,000 participants per trial to reduce random sampling variability and thus clearly illustrate bias. The data were generated under the following more general model:(2)

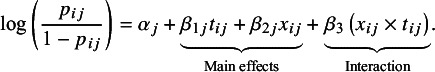



This differs from Model (1) by allowing for potentially different and non-exchangeable main effects of the treatment ( 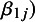 or prognostic effect of the covariate ( 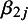 ) as detailed in the following subsections and Supplementary Table S1. In Scenarios 1–3, the data-generating mechanism does not include an individual-level treatment–covariate interaction within any study ( 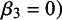 , while Scenario 4 demonstrates a scenario with true effect modification ( 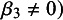 . We note that these hypothetical scenarios use high degrees of heterogeneity in the treatment effects, covariate distributions, and allocation ratios to illustrate the potential for bias despite the use of IPD and randomization within each trial; real-data examples of confounding will likely be less extreme.

### 4.3 Scenario 1: No treatment–covariate interaction and no trial-level confounding

In Scenario 1, 50% of the participants in each trial are seropositive at baseline, and each trial has a 1:1 allocation ratio of treatment to placebo. The prognostic effect of the covariate is constant across trials (OR = 0.5), so the trial-level treatment effects are generated as independent of both the allocation ratio and 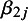 . As described above, there is no individual-level interaction between the treatment and covariate within any of the trials ( 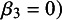 . Figure 2a shows the estimated interactions within each study (dotted lines), based on separate trial-specific logistic regression models, and the pooled interaction (solid line) under Model (1). The points and diamonds represent the trial-specific and pooled covariate-specific treatment effects, respectively, with sizes proportional to the inverse of their variances. Here, the trial-specific estimates and the pooled estimate of the interaction between treatment and baseline serostatus from Model (1) are consistent. None indicates that the presence of anti-SARS-CoV-2 antibodies at baseline modifies the treatment effect.

### 4.4 Scenario 2: Association between trial-level treatment effect and proportion seropositive

We now demonstrate how confounding can arise due to the association of the trial-level treatment effect with the distribution of the covariate within each trial, in this case the proportion of participants seropositive at baseline. Suppose that the seropositivity rate varies from 10% to 70%; this could arise from an increased prevalence of previous infection or vaccination over time as was observed during the COVID-19 pandemic. Furthermore, suppose the emergence of vaccines and increase in previous infections coincided with a decrease in the treatment effects of passive immunotherapies designed earlier in the pandemic due to viral mutations.^29^ Under this setting (Scenario 2), there is a correlation between the trial-level seropositivity rate and the treatment effect due to trial-level confounding.

Figure 2b shows how, under this scenario, when using Model (1), which assumes common underlying effects across trials, the pooled interaction estimates under the model given above differ from the interaction estimates within a trial. Specifically, while there is no evidence of any interaction when trials are analyzed separately, the meta-analysis suggests that the treatment is more effective among participants who are seronegative at baseline. This counterintuitive result arises because there were larger treatment effects in trials with more seronegative participants, pulling the estimated log odds ratio in that subgroup away from 0, while there were smaller treatment effects in trials with more seropositive participants, pulling the estimate in that subgroup towards 0, resulting in an apparent but artificial interaction (Figure 2b).

### 4.5 Scenario 3: Association between trial-level prognostic effect of seropositivity and treatment allocation ratio

The estimated interaction between the treatment assignment and baseline serostatus can be interpreted as the difference in the association between baseline serostatus and mortality between participants assigned to treatment or placebo. Figure 2c shows the prognostic effect of baseline serostatus by treatment group for Scenario 1. Like Figure 2a, neither the trial-specific nor pooled results based on within-trial information show evidence of an interaction. Specifically, while participants seronegative at baseline had higher odds of mortality in all but one trial (log OR = 0), this did not differ by treatment group within a trial or when pooled across trials. However, inconsistency between these results can arise if the allocation ratio to treatment or placebo differs across trials and is correlated with the main (prognostic) effect of baseline serostatus. For example, the allocation ratio to treatment versus placebo could have increased over time as additional agents were added to the TICO/ACTIV-3 platform. This could coincide with the prognostic effect of baseline serostatus waning due to the antibody assay used being specific to previous strains of the virus. Thus, in Scenario 3, the proportion in the treatment group varies from 10% to 70% and is correlated with the prognostic effect of the covariate. To isolate the role of the prognostic effect, none of the trials have an overall treatment effect (OR = 1) nor any treatment effect modification. Figure 2d demonstrates how this phenomenon would again result in a positive slope for the estimated interaction and thus larger estimated treatment effect among seronegative participants, despite no evidence of this within any given trial.

### 4.6 Supplemental Scenario 4: True effect modification and no trial-level confounding

Finally, we also include a supplemental scenario identical to Scenario 1 (no trial-level confounding), but with true individual-level effect modification. Suppose, as hypothesized a priori in the ACTIV-3/TICO platform, the effect of treatment was greater among those without SARS-CoV-2 neutralizing antibodies at baseline. Specifically, we generate data where each trial has a ratio of odds ratios (exp[ 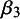 ]) comparing seropositive to seronegative participants of 0.8. Supplementary Figure S1 shows that, as in Scenario 1, the estimated pooled interaction from Model (1) is consistent with those within each trial, though in this case, each of these shows a greater treatment effect among seronegative participants.

**Figure 3:**
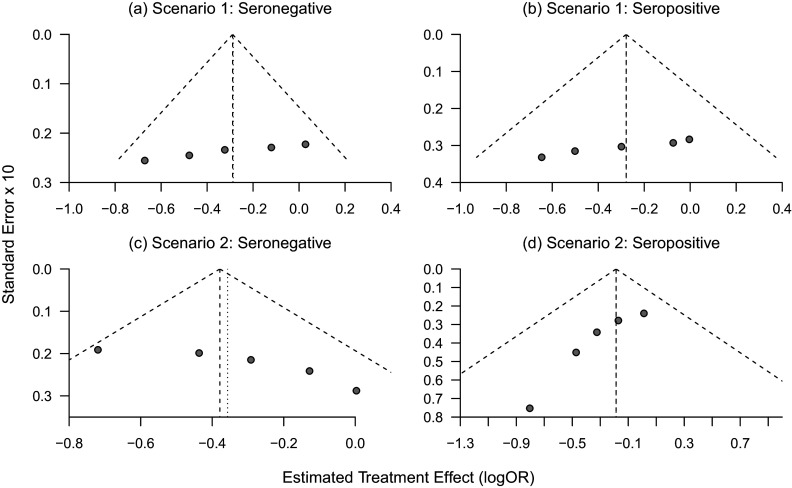
(a–d) Funnel plots of trial-specific treatment effects for seronegative and seropositive participants under Scenario 1 (a–b; no trial-level confounding) and Scenario 2 (c–d; trial-level confounding by seropositivity).

## 5 Correspondence with funnel plot asymmetry

Funnel plots display a scatter plot of study or trial-specific effect estimates, typically against some measure of each trial’s weight, such as the precision or sample size.^30^^,^ ^31^ If the effect estimates are exchangeable with respect to this measure, the scatterplot will resemble a symmetric inverted funnel, where trials with smaller weights and greater uncertainty have a larger spread at the bottom of the plot, and trials with larger weights and greater precision converge at the top of the plot.^32^ Funnel plot asymmetry can arise due to publication bias, where, for example, trials with null effects are less likely to be published, but it can also arise due to other sources of heterogeneity.^32^ Here, we illustrate how trial-level confounding can be observed as funnel plot asymmetry.

In Scenario 2, the proportion of seropositive participants in each trial, and thus the weight this subgroup contributes to the pooled analysis, is correlated with the subgroup-specific treatment effects across trials. This source of trial-level confounding can, thus, be directly seen by fitting separate models for subgroup within each trial and generating subgroup-specific funnel plots. Figure 3a and 3b, generated using the R package “meta,”^33^ shows the relationship between the estimated trial-specific treatment effects and their standard errors separately among seronegative and seropositive participants in each trial, under Scenario 1 where the truth is that the treatment effects are unrelated to the rate of antibody positivity. Figure 3c and 3d shows these estimates under Scenario 2 where the trial-level treatment effects were correlated with the proportion of participants seropositive at baseline, which led to aggregation bias when pooling across trials when using Model (1). While there are only five trials, Figure 3a and 3b looks approximately symmetric, while Figure 3c in particular, shows clear asymmetry. Furthermore, Figure 3c and 3d shows opposite patterns, due to the trials with larger treatment effects having more seronegative participants (and thus lower standard errors), with the opposite pattern being true for the seropositive subgroup. This corresponds to the right-hand side of Figure 2b, where trials with more seropositive participants are pulling the estimated treatment effect in that subgroup toward the null. Supplementary Figure S2 shows subgroup-specific funnel plots for Scenario 4, where there is true effect modification. This shows a similar pattern within each subgroup as Scenario 1, as the data-generating mechanisms differ only by a constant difference in the logORs across subgroups in Scenario 4. We do not display funnel plots for Scenario 3 as each of the estimated log ORs for the treatment effect are approximately zero, consistent with the data-generating mechanism. Scenario 3 results in aggregation bias due to non-exchangeability of the prognostic effects of the covariate across trials; this could be seen in funnel plots of the prognostic effects of the covariate by treatment group.

Despite the clear connection between funnel plot asymmetry and potential for trial-level confounding, tests for asymmetry are often underpowered^32^ and should not be solely relied on to assess for potential trial-level confounding. This approach still applies when some trials do not recruit participants across all subgroups of interest. We also note that the covariate in this example is categorical; for a continuous covariate, one could inspect funnel plots after discretizing the variable into categories. Alternatively, one could instead plot and compare the within and between-trial relationships as shown in Figure 1. However, while funnel plots could play a role in detecting trial-level confounding that may lead to aggregation bias, most importantly, this connection emphasizes how aggregation bias in this setting arises from non-exchangeability of the subgroup-specific treatment effects across trials. Thus, when trial-level confounding may be present, one should carefully consider the interpretability of the pooled estimate.

## 6 Avoiding bias due to trial-level confounding through stratification

The trial-level confounding described above arises due to assumptions about whether trial-specific main effects are exchangeable or differ systematically with the distribution of the covariate or the allocation ratio. In this case, the main effects serve as an “anchor,” causing the pooled estimate of the interaction to be influenced by the trend in main effects across trials (across-trial information). Thus, to avoid the counterintuitive results shown in Figure 2, one can remove assumptions about how these main effects relate to each other through stratification of all parameters (except the interaction term) by trial as follows^3^^,^ ^34^:

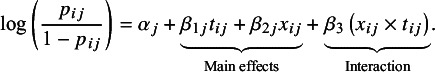



Here, 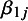 refers to the main effect of treatment in the trial 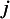 (e.g., treatment effect among seronegative participants), while 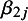 represents the prognostic effect of the covariate (baseline serostatus) in the trial 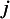 . This ensures that 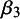 corresponds solely to the treatment–covariate interaction at the individual level, based on within-trial information (thus avoiding trial-level confounding). This model (Model (2)) is the same as that in the data generating mechanism for the simulated scenarios described above.

**Table 1:** *Estimated interaction coefficients (* 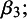 *[95% CIs]) from common (fixed) effect meta-analysis for a single simulated dataset under three scenarios, fit with the following models: (1) one-stage logistic regression models using between-study information, (2) two-stage multivariate meta-analysis of interaction and main effects, (3) one-stage logistic regression model with only within-study information by stratification of main effects, (4) one-stage logistic regression model separating out within-study and across-study information, by centering covariate and including an interaction between treatment and the study mean, and (5) two-stage meta-analysis of only within-trial interaction coefficients*

		Scenario 1	Scenario 2	Scenario 3
		No trial-level	Confounding by	Confounding by
		confounding	covariates	allocation ratio
Between-study information	(1) One-stage	0.01 [−0.02, 0.04]; *p* = 0.56	0.19 [0.16, 0.23]; *p* < 0.01	0.20 [0.17, 0.23]; *p* < 0.01
	(2) Two-stage multivariate meta-analysis	0.01 [−0.03, 0.04]; *p* = 0.63	0.19 [0.15, 0.22]; *p* < 0.01	0.20 [0.17, 0.23]; *p* < 0.01
Within-study information only	(3) One-stage (stratification)	0.01 [−0.03, 0.04]; *p* = 0.67	−0.03 [−0.07, 0.01]; *p* = 0.15	0.00 [−0.03, 0.03]; *p* > 0.99
	(4) One-stage (centering covariate)	0.01 [−0.02, 0.04]; *p* = 0.56	−0.03 [−0.07, 0.01]; *p* = 0.11	0.00 [−0.03, 0.03]; *p* > 0.99
	(5) Two-stage; only interaction coefficients	0.01 [−0.03, 0.04]; *p* = 0.67	−0.03 [−0.07, 0.01]; p = 0.15	0.00 [−0.03, 0.03]; *p* > 0.99

Alternatively, one could center the covariate by the trial-specific mean and include an interaction between the trial mean and the main effect of treatment as an additional term^3^:(3)

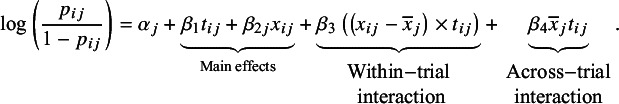



The primary distinction between Models (2) and (3) is that the latter still allows the main effect of treatment to differ across trials but assumes a linear relationship with the trial mean. Specifically, in Model (3), the main effect of treatment is equal to 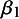 + 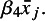 Centering of the covariate within each trial is done to remove most of the dependence between 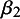 , 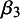 , and 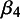 . We note that this model still stratifies the main (prognostic) effect of the covariate ( 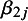 ) by trial. Random effects on the two main effects ( 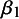 and 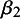 ) are also possible under Model (3).

Another option is a two-stage IPDMA, pooling only the within-trials interaction coefficients in a univariate meta-analysis. The first stage automatically stratifies intercepts and main effects by trial, and so the second stage avoids assumptions about how the trial-specific treatments effects are associated with the aggregated covariate values. Thus, the meta-analysis summarizes the within-study interaction without any confounding due to between-study information. We note that a two-stage multivariate meta-analysis approach pooling the coefficients for the main effects and interaction term jointly would not be equivalent,^3^ as this typically assumes that the main effects are the same (fixed/common effect) or exchangeable (random effects). In particular, this model is approximately equivalent to Model (1) when using the same model assumptions (e.g., random vs. common effects) and thus has the same potential for trial-level confounding as described above and elsewhere.^3^^,^ ^11^^,^ ^35^

Table 1 shows the results of analyzing each scenario using each of the above one- and two-stage approaches with and without separation of within- and between-trial information (i.e., using Models (1), (2), and (3)). Models for Scenarios 1 and 3 centering the covariate do not include the interaction term between the treatment effect and trial mean (proportion of seropositive participants), as this is constant (50%) across trials. Scenario 1 has no trial-level confounding, and all methods result in an estimated interaction slope of 0.01, with nearly identical 95% confidence intervals. Supplementary Table S2 also shows that under Scenario 4 (no trial-level confounding, true treatment effect modification) each of the methods give identical results ( 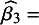 −0.23 [−0.26, −0.19]; *p* < 0.01) that are close to the true value. However, using across-trial information when analyzing Scenarios 2 and 3 in either a one-stage or two-stage model results in a statistically significant interaction, corresponding to a greater treatment effect among individuals seronegative at baseline. Thus, Model (1), whether fit using a one-stage or two-stage approach yields biased estimates in Scenarios 2 and 3. None of the models that stratify the main effects, either when using a one-stage approach or a two-stage approach, show evidence of this, as they avoid allowing the across-trial associations to influence the estimated interaction, thus avoiding this bias. Supplementary Table S3 shows the results when using random effects for 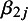 and 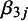 rather than stratifying these terms; in this case, this model gives similar results to the stratified results for all scenarios, likely because the trial-specific main effects are allowed to differ. However, this model can incorporate between-trial information due to shrinkage of the random trial-specific main effects toward each other, causing bias if the subgroup-specific main effects are not exchangeable. This issue is similar to that previously described in arm-based network meta-analysis, where random study-specific intercepts can introduce bias due to incorporating between-study information through shrinkage.^36^^,^ ^37^ The simulated datasets for each trial in all four scenarios above are very large (*n* = 100,000 each) and have overall treatment effects spread evenly about the overall mean, likely making these issues less of a concern in this setting. A further challenge is whether to account for the likely correlation amongst the random effects; this correlation can be difficult to estimate (non-convergence) and might inflate the issue of contamination between within and across-trial relationships.

## 7 Estimating covariate-specific treatment effects

In Model (1), without stratification, the estimated ORs representing the conditional treatment effects in participants seronegative and seropositive at baseline would be 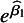 and 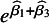 , respectively. However, when stratifying the main effect of treatment (either naturally via the two-stage approach, or within the one-stage model), it is not immediately apparent how to estimate pooled covariate-specific treatment effects, since 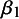 is estimated separately for each trial. Possibly as a result, most previous approaches to estimating covariate-specific treatment effects from IPDMA do not separate within- and across-trial information and are thus vulnerable to trial-level confounding. These include a multivariate meta-analysis of the main effect of treatment and the interaction estimates,^35^ a “metacurve” approach, where covariate-specific treatment effect curves are first estimated within each trial and then pooled in a pointwise fashion,^38^ or a one-stage approach like Model (1). These each assume exchangeability of the covariate-specific treatment effects, leaving open the possibility of trial-level confounding as demonstrated above.

**Figure 4:**
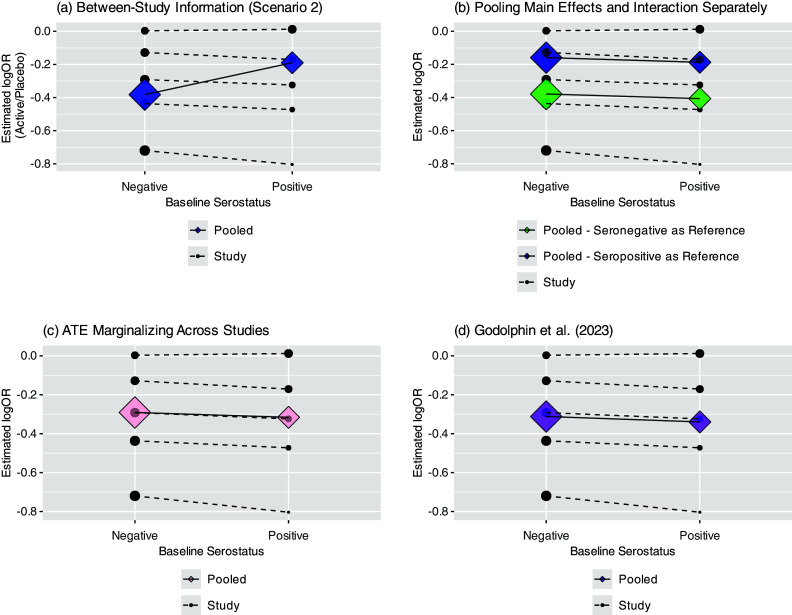
(a–d) Four panels showing pooled subgroup-specific treatment effects from Scenario 2 (a) using between-study information; (b) when pooling interaction and main effects separately (Riley et al.; results depend on reference level); (c) when estimating the average treatment effect (ATE) marginalized across studies and (d) using Godolphin et al. approach (reweighting).

Several approaches have been proposed to estimate covariate-specific treatment effects in a within-study framework, thus avoiding study-level confounding. Riley et al.^3^ suggested pooling the main effect of treatment and the interaction terms separately, thus avoiding the incorporation of across-trial information into the estimate of the interaction. However, doing so anchors the estimate of the covariate-specific treatment effects on the estimated treatment effect at the reference level, and the pooled subgroup-specific treatment effects then depend on the selected reference group. Figure 4b demonstrates this phenomenon; the subgroup-specific treatment effects clearly differ based on whether the seronegative or seropositive group is chosen as the reference. Deriving confidence intervals for these covariate-specific treatment effects would also likely require a bootstrap or other similar procedure.

Alternatively, if using a one-stage approach, one could fit a stratified model and then estimate marginal effects, such as the estimated average treatment effect marginalized over the empirical distribution of trial assignment.^11^ This involves estimating the treatment effect for each participant by first comparing the fitted values under assignment to treatment or control (and a specific subgroup), then averaging across participants to estimate the average treatment effect (ATE). Figure 4c shows this using the R package “marginaleffects.”^39^ Godolphin et al.^4^ also proposed a framework for estimating subgroup-specific treatment effects within a two-stage approach by including a constraint that the difference between the subgroup-specific treatment effects is equal to the pooled interaction coefficient based only on within-trial information (Figure 4d). This leads to a reweighting of the subgroup-specific effects within each trial, avoiding aggregation bias when estimating the pooled (or “floating”) subgroup-specific effects.^4^ In Scenario 2, this gives very similar results to marginalizing when using a one-stage model with stratification of the main effects. However, work extending this framework to continuous covariates is ongoing. Finally, as mentioned above, rather than stratifying the main effect, one could center the covariate by its trial-specific means as in Model (3) and estimate covariate-specific treatment effects conditional on the study mean. However, this may not be sensible if there is no trial-level confounding, and like tests of funnel plot asymmetry, the test of the effect of the trial mean (across-trial interaction) will often be underpowered.^12^

## 8 Sensitivity analysis

While some options exist for estimating covariate-specific treatment effects using only within-trial information, each has limitations; estimating covariate-specific treatment effects is still more straightforward without stratifying the main effect of treatment. In addition, while there is a potential for trial-level confounding in any IPDMA of treatment–covariate interactions, this may not always be present or meaningfully impact conclusions. It has also been noted that estimating the interaction based only on within-trial information could result in a loss of power^12^^,^ ^16^; we hypothesize this may be particularly true if the model contains many treatment effect terms, such as when using splines to model a non-linear association with a continuous covariate. Riley et al.^11^ suggest estimating the degree of aggregation bias directly as the difference between the within and across-trial interaction covariates in Model (3). However, this quantity frequently has a high degree of uncertainty and does not directly represent the influence of the across-study association on the pooled interaction estimate. This is because the pooled interaction reflects a weighted average of the within- and across-trial relationships when these are not disentangled.^11^ When there is a high degree of variability in the estimated across-trial association, such as when the covariate means for each trial are similar or the number of trials is small, this may have little weight in the combined estimate.

Instead, to directly assess the robustness of results to potential trial-level confounding, one may fit models with and without stratification of the main effect of treatment, while always stratifying the covariate main effect when possible. In the case that the point estimates for the interaction term are qualitatively similar between models, we propose proceeding with the model without stratification, as the results are unlikely to be impacted by trial-level confounding. If the results differ to a clinically meaningful degree, one might then further investigate potential trial-level confounding using funnel plots and comparisons of the covariate distributions across studies. If the covariate-specific treatment effects are not exchangeable across trials (trial-level confounding present), the pooled results may lack interpretability, particularly if using a random effects model. However, if subgroup-specific treatment effects were still considered meaningful, one could stratify all main effects in the model and marginalize over the trials, estimate these conditional on the trial mean, or proceed with the reweighting approach proposed by Godolphin et al.^4^

Differences between the estimated covariates across models unrelated to trial-level confounding are possible when the outcome is binary, due to the non-collapsibility of the odds ratio.^40^ However, estimating the aggregation bias directly as the difference between within- and across-trial associations as proposed by Riley et al.^11^ as well as the approach of testing the equivalence of these two terms^11^^,^ ^12^^,^ ^16^ have this same limitation, as the within-trial interaction and across-trial interaction (with the trial mean) may not be equivalent in this setting. Furthermore, the goal of the proposed sensitivity analysis is to determine the sensitivity of conclusions to the choice of model, rather than to assume equivalence between these quantities.

**Figure 5:**
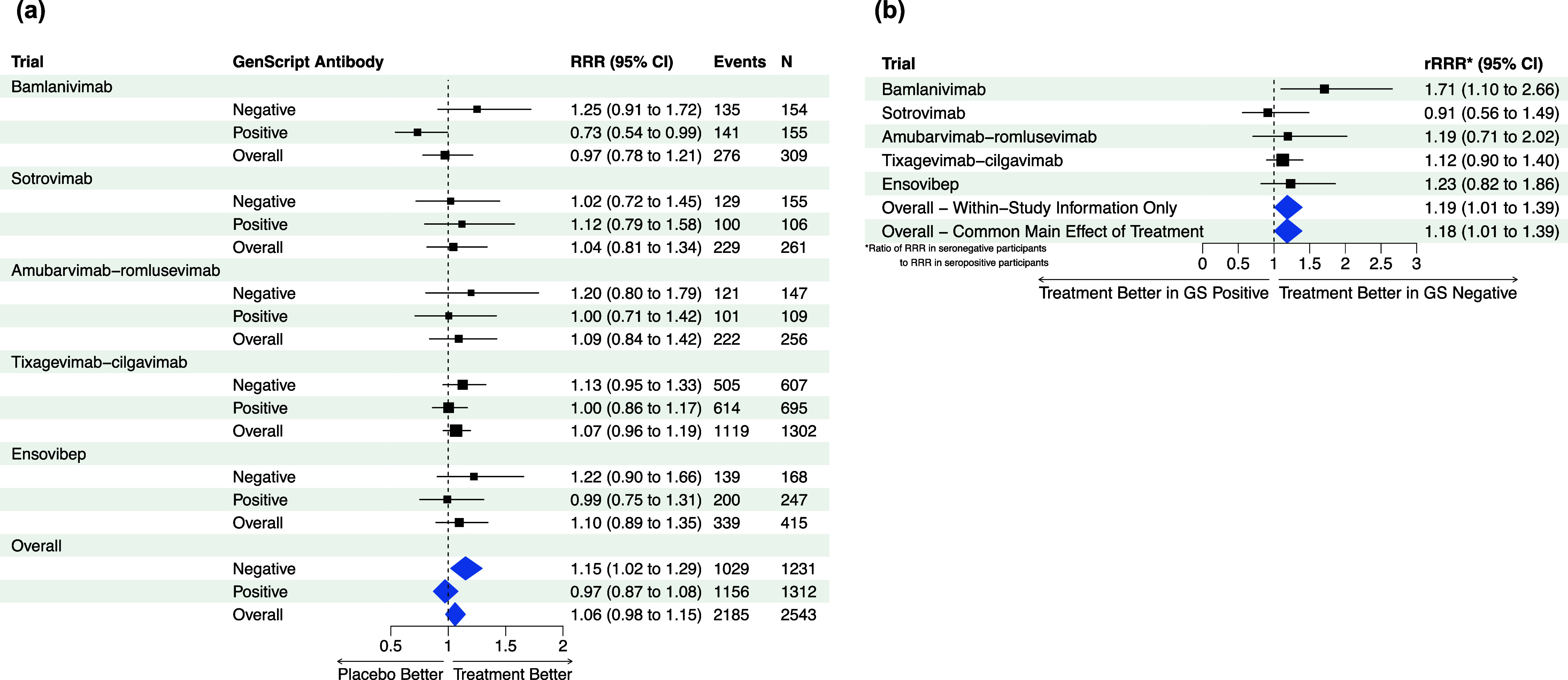
(a–b; TICO/ACTIV-3) (a) Estimated study-specific effects of passive immunotherapy on time to sustained recovery overall and by baseline serostatus without stratification of main effect of treatment; (b) study-specific interaction estimates and pooled interaction estimates with and without stratification of main effect of treatment.

## 9 Application to ACTIV-3/TICO

Figure 5a shows the estimated recovery rate ratio (RRR) for time to sustained recovery in each of the five ACTIV-3/TICO trials and the pooled result overall and by baseline serostatus. The subgroup-specific treatment effects are estimated from Fine–Gray models fit within each study, accounting for the competing risk of death, with an indicator of treatment assignment, indicator of baseline antibody positivity, and a treatment–serostatus interaction, using the R package “cmprsk.”^41^ The pooled results are obtained from a similar one-stage Fine–Gray model with stratification of the main (prognostic) effects of serostatus by study and a shared treatment effect. Supplementary Figure S3 shows funnel plots of the trial-specific estimated log(RRRs) within the seronegative and seropositive subgroups; neither display evidence of asymmetry.

We note this model does not fully separate within and between-study information, but prefer a model with a single treatment effect across trials as opposed to separate unrelated effects for interpretation. Thus, following the sensitivity analysis approach proposed above, Figure 5b shows the within-study results for the treatment–serostatus interaction (ratio of RRRs) within each study, overall based on within-study information only (by stratifying all main effects), and from the model with the shared treatment effect. The estimated ratios of RRRs (seronegative/seropositive) are nearly identical with stratification (rRRR: 1.19, 95% CI: 1.01, 1.39) and without stratification of the main effect of treatment (rRRR: 1.18, 95% CI: 1.01, 1.39). Supplementary Table S4 also shows the results under the full set of models fit on the simulated data; the results are highly consistent across models, with the point estimates of the rRRR ranging from 1.17 to 1.19. We conclude that the observed interaction between the effect of the treatment and baseline serostatus is unlikely to be a result of study-level confounding and proceed with the previous model that allows straightforward estimation of the subgroup-specific treatment effects. This indicates no evidence of treatment benefit overall or among participants positive for anti-SARS-CoV-2 antibodies at baseline, but a significant treatment effect among participants without antibodies at baseline (RRR: 1.15, 95% CI: 1.02, 1.29).

## 10 Conclusion

Aggregation bias can occur in a meta-analysis of treatment–covariate interactions, despite randomization of treatment assignment within each trial, when within-trial and across-trial information is combined. Framing aggregation bias as trial-level confounding that arises when trial treatment effects are related to within-trial covariate distributions, despite no actual treatment effect modification, may be more intuitive than focusing on the distinction between within- and across-trial information. Existing tools can be used to identify study-level confounding; funnel plots reflect whether treatment effects appear to be exchangeable with respect to study weights, while a sensitivity analysis can be used to assess the robustness of conclusions to study-level confounding.

In summary, greater intuition about potential sources of aggregation bias and a simple sensitivity analysis to identify meta-analyses at high risk of trial-level confounding can improve the methodological quality and robustness of future meta-analyses of treatment–covariate interactions.

## Supporting information

Siegel et al. supplementary material Siegel et al. supplementary material

## Data Availability

The data from TICO/ACTIV-3 are publicly available at: https://public-data.ccbr.umn.edu/, and the R code is available for download at: https://github.com/liannesiegel/TrialLevelConfounding.

## Supplementary material

To view supplementary material for this article, please visit http://doi.org/10.1017/rsm.2025.10043.
