# Supplementary material for: Examining covariate-specific treatment effects in individual participant data meta-analysis: Framing aggregation bias in terms of trial-level confounding and funnel plots
Source: Res Synth Methods. 2025 Oct 23;17(1):194–209. doi: 10.1017/rsm.2025.10043 (PMC12823212; doi:10.1017/rsm.2025.10043)
Supplement: Siegel et al. supplementary material [file S1759287925100434sup001.docx]

**Supplementary Material**

Trial-level confounding when examining treatment effect heterogeneity in individual participant data meta-analysis of randomized clinical trials

Lianne K. Siegel, PhD^1^, Joseph S. Koopmeiners, PhD^1^, Jamie Hartmann-Boyce, PhD^2^, Peter J. Godolphin, PhD^3^, Abdel G. Babiker, PhD^3^, Giota Touloumi, PhD^4^, Kirk U. Knowlton, MD^5^, Richard D. Riley, PhD^6^

^1^Division of Biostatistics and Health Data Science, School of Public Health, University of Minnesota, Minneapolis, MN, USA

^2^Department of Health Promotion and Policy, School of Public Health & Health Sciences, University of Massachusetts Amherst, Amherst, MA, USA

^3^MRC Clinical Trials Unit at University College London, London, UK

^4^Department of Hygiene, Epidemiology and Medical Statistics, Medical School, National and Kapodistrian University of Athens, Athens, GR

^5^Intermountain Medical Center, Intermountain Heart Institute, Salt Lake City, UT, USA

^6^School of Health Sciences, University of Birmingham, Birmingham, UK

# **Supplementary Tables**

# **Table S1:** Data generation mechanisms for Scenarios 1-4.

|  | Scenario 1 | Scenario 2 | Scenario 3 | Scenario 4 |
| --- | --- | --- | --- | --- |
| Number of trials | 5 | 5 | 5 | 5 |
| Participants per trial | 100,000 | 100,000 | 100,000 | 100,000 |
| % Mortality among seronegative participants in control arm | 20% | 20% | 20% | 20% |
| % Assigned to treatment | 50% | 50% | For studies 1-5:  10%, 25%, 40%, 55%, 70% | 50% |
| % Antibody positive at baseline | 50% | For studies 1-5:  10%, 25%, 40%, 55%, 70% | 50% | 50% |
| ${\exp(\beta}_{1j}):$treatment effect in seronegative subgroup* (OR; Active/Placebo) | For studies 1-5:  OR = 0.5, 0.625, 0.75, 0.875, 1 | For studies 1-5:  OR = 0.5, 0.625, 0.75, 0.875, 1 | 1 (No treatment effect) | For studies 1-5:  OR = 0.5, 0.625, 0.75, 0.875, 1 |
| exp($\beta_{2j}):$covariate prognostic effect (OR; Seropositive/Seronegative) | OR = 0.5 | OR = 0.5 | For studies 1-5:  0.5, 0.625, 0.75, 0.875, 1 | OR = 0.5 |
| $\beta_{3}:$ interaction coefficient comparing treatment effect among seropositive vs. seronegative participants | 0 | 0 | 0 | log(0.8) ≈ -0.22 |

*Treatment effect in all participants when no interaction/effect modification ($\beta_{3}$ = 0)

**Table S2**: Estimated interaction coefficients ($\beta_{3};$[95% CIs]) from common (fixed) effect meta-analysis under supplemental Scenario 4, fit with the following models: (1) one-stage logistic regression models using between-study information, (2) two-stage multivariate meta-analysis of interaction and main effects, (3) one-stage logistic regression model with only within-study information by stratification of main effects, (4) one-stage logistic regression model separating out within-study and across-study information, by centering covariate and including an interaction between treatment and the study mean, and (5) two-stage meta-analysis of only within-trial interaction coefficients.

|  |  | **Scenario 4**  True Effect Modification, No Trial-Level Confounding |
| --- | --- | --- |
| Between-Study Information | (1) One-stage | -0.23 [-0.26, -0.19]; p < 0.01 |
|  | (2) Two-stage multivariate meta-analysis | -0.23 [-0.26, -0.19]; p < 0.01 |
| Within-Study Information Only | (3) One-stage (Stratification) | -0.23 [-0.26, -0.19]; p < 0.01 |
|  | (4) One-stage (Centering Covariate) | -0.23 [-0.26, -0.19]; p < 0.01 |
|  | (5) Two-stage; only interaction coefficients | -0.23 [-0.26, -0.19]; p < 0.01 |

**Table S3:** Estimated interaction coefficients ($\beta_{3};$[95% CIs]) for Scenarios 1-4 under one-stage logistic regression models similar to model (1) but with random main effects for the treatment and covariate. These models still incorporate some between-trial information due to shrinkage of the trial-specific coefficients.

|  |  | **Scenario 1**  No Trial-level Confounding | **Scenario 2**  Confounding by Covariate Distribution | **Scenario 3**  Confounding by Allocation Ratio | **Scenario 4**  True effect Modification  (No Confounding) |
| --- | --- | --- | --- | --- | --- |
| Between-Study Information | One-stage:  random main effects ($\beta_{1j}, \beta_{2j});$  common/fixed $\beta_{3}$ | 0.00 [-0.31, 0.31]; p = 0.87 | -0.03 [-0.06, 0.01]; p = 0.15 | 0.00 [-0.03, 0.03]; p = 0.94 | -0.23 [-0.26, -0.20];  p < 0.01 |

**Table S4:** Estimated ratios of recovery rate ratios (rRRRs) comparing the treatment effect among seronegative vs seropositive participants in TICO/ACTIV-3 for all models

|  |  | **Estimate** | **95% CI** |
| --- | --- | --- | --- |
| Within-study information only | Fully stratified (model 2) | 1.19 | [1.01, 1.39] |
|  | Centered (model 3) | 1.18 | [1.01, 1.39] |
|  | Univariate meta-analysis | 1.18 | [1.01, 1.39] |
| Between-study information | Shared treatment effect | 1.18 | [1.01, 1.39] |
|  | No stratification (model 1) | 1.17 | [0.99, 1.37] |
|  | Multivariate meta-analysis | 1.17 | [1.00, 1.37] |

**Supplementary Figures**

**Figure S1:** Scenario 4 (no trial-level confounding and true effect modification). Solid dots represent estimated log OR comparing treatment vs placebo by baseline serostatus in each trial and diamonds represent estimated subgroup-specific pooled effects from model (1). Within-study and pooled estimated interactions given by dashed and solid lines, respectively.

**Figure S2:** Funnel plots of trial-specific treatment effects for seronegative and seropositive participants under Scenario 4 (effect modification and no trial-level confounding).

**Figure S3:** Funnel plots of estimated trial-specific treatment effects (RRRs) for seronegative and seropositive participants in TICO/ACTIV-3.
